# Prediction of first marriage age in China and factors associated with marriage delay: Analysis of the 2020 national census data

**DOI:** 10.1371/journal.pone.0338259

**Published:** 2025-12-29

**Authors:** Qihang Zhao, Dongxia Zhao

**Affiliations:** 1 China Center for Health Development Studies, Peking University, Beijing, China; 2 School of Geographical sciences, Liaoning Normal University, Dalian, Liaoning, China; University of Salamanca, SPAIN

## Abstract

China is undergoing the second demographic transition. This paper constructs provincial-level Net nuptiality tables by gender and urban-rural residence using national census data. We compare spatial patterns of expected years in the single state among Chinese subpopulations and analyze determinants of marriage delay. Results indicate a consistent postponement of first marriage. In 2020, the expected duration of singlehood since birth reached 33.63 years for males and 29.56 years for females. Urban males and rural females exhibited tendencies toward earlier marriage. Key determinants delaying first marriage included educational attainment and the proportion of ethnic minorities, which exerted antagonistic effects. The postponement effect of education outweighed the inhibitory influence of ethnic cultural factors, collectively driving the sustained rise in China’s age at first marriage.

## Introduction

In 1987, Van de Kaa [1] conducted a study on countries that completed the demographic transition in the mid-20th century. Notably, the demographic and family dynamics of Nordic and Western European countries have experienced significant changes. These changes were observed in the form of delayed marriage, higher divorce rates, increased cohabitation, and extramarital fertility rates. On the basis of these emerging trends, Van der Kaa proposed the concept of the “second demographic transition” to predict overall demographic development following classical demographic transition theory. Presently, China is undergoing a noticeable shift in the age of first marriage, signifying the onset of the “second demographic transition.” Data from successive China population censuses reveal that the average age of first marriage for men has risen from 24.72 years in 1980 to 29.38 years in 2020. Similarly, the average age at first marriage for women has increased from 22.88 years to 27.95 years. These figures demonstrate a significant delay in the average age of first marriage among young people in China over the past four decades. This trend contradicts the traditional norms of early and universal marriage that have long been upheld in China [2].

China's cultural background and intergenerational family transition have maintained marriage as a significant event in the lives of its residents [3]. However, socioeconomic changes, such as the implementation of compulsory education, expanded tertiary education, the impact of the one-child policy, and the aging population, have led to a shift in attitudes toward marriage and family among Chinese youth [4–6]. Most studies have focused only on single indicators, such as marriage intentions or divorce rates, when examining factors contributing to the postponement of marriage. This approach fails to provide a comprehensive understanding of the underlying causes and hinders accurate evaluations of trends such as “non-marriageism” and delayed marriages among youth. Consequently, it affects the formulation of marriage policies, fertility policies, and decisions related to family functioning. Consequently, examining the expected years in the single state since birth among Chinese youth and analyzing the drivers of marriage postponement is critical, particularly amidst the shifting population age structure.

First, although the marriage concept and behavior of Chinese youth groups have undergone changes, statistical data from 2000--2017 show that the proportion of individuals aged 50 years and above who have not married in China is less than 2% [7], indicating a very low level of non-marriage in this age group. This suggests that the majority of youth in China do not abstain from marriage entirely but rather delay it. Therefore, it becomes crucial to identify the key drivers behind the increasing delay in the age at which individuals marry.

China, as a multi-ethnic country, has varying levels of economic and social development across its eastern, central, and western regions, as well as significant regional cultural differences. For example, research has shown that the average age at first marriage among ethnic minority groups in Southwest China tends to be earlier than that of the Han Chinese population in the same region, with both males and females exhibiting similar trends [8]. This phenomenon implies that the delay in age at first marriage, which is observed across China as a whole, is profoundly influenced by regional cultural differences, indicating considerable regional cultural heterogeneity. Consequently, it is crucial to explore whether there is a regional effect on the postponement of marriage, specifically in relation to different genders, under the influence of ethnic cultural differences. Furthermore, it is essential to examine whether there is a periodization of the effect of these ethnic cultural differences on the age at first marriage in China.

Second, it is possible to approximate the tendency of young people in a specific region to marry early or late by calculating the average age at first marriage over multiple time periods. However, there are factors that can disrupt this indicator, such as the age structure of the population and mortality risk. For example, if a region has a predominantly younger age structure, the average age at first marriage may also appear younger, and vice versa [9–12]. Conversely, this relationship also holds true. Therefore, comparing the average age at first marriage between different regions or countries can often lead to fallacies and distortions in the research findings due to variations in population factors. To mitigate the influence of population age structure and mortality risk on the calculation of age at first marriage in a universal context, it is necessary to focus research on the probability of this group being exposed to the risk of death. This approach enables more realistic estimation of expected years in the single state since birth among Chinese. Consequently, it is imperative to investigate emerging trends in China's first-marriage patterns after controlling for population age structure and mortality risk, with particular focus on temporal shifts in marriage timing as reflected in age-specific first-marriage probabilities and proportions never-married within the cohort since 2000, while also examining provincial variations in first-marriage dynamics.

Third, China's policy of expanding university education, which was initiated in 1999, has had a gradual impact on the educational structure of the population [4,13]. This expansion has significantly increased the proportion of educated individuals and the number of years of education attained by the Chinese population. Consequently, this structural shift has led to a delay in the entry of China's youth into the workforce and hindered capital accumulation. Consequently, the age at which young people complete their first marriages has been significantly delayed. A similar trend was observed in American society approximately 1950 [14]. Given that China remains a society with prevalent universal marriage norms, it is reasonable to assume that the postponement factor associated with shifting marriage concepts among youth is significantly amplified. Therefore, it is crucial to address the issue of delayed progression in various life stages, starting from education to the completion of the first marriage. Consequently, a thorough analysis of the average number of years of education, while considering regional and period effects, is warranted. This analysis should investigate the extent to which the postponement of first marriages in contemporary China is influenced by years of education as the primary driving factor. Moreover, it is important to account for regional and period effects and explore the potential moderating effects of years of schooling across different regions.

In addition, there are some novel findings in this paper that further supplement the shortcomings of previous research areas. First, through the calculation of the multi-decreasing first marriage table, we find that the trend of delaying the first marriage of young people in all provinces of China in 2020 is further increasing, the spatial and temporal differences in the age of first marriage are gradually widening, and the gender differences in the age of first marriage between urban and rural areas show diametrically opposed characteristics. For example, the age range for young men to complete their first marriages is concentrated and short, while the age range for women to complete their first marriages is dispersed and long-lasting; men in urban areas and women in rural areas complete their first marriages at an earlier age, while women in economically developed areas and men in economically backward rural areas complete their first marriages at a later age. Second, we empirically test the effects of educational attainment and ethnic and cultural differences on delayed age at first marriage, controlling for time and region, for the Chinese youth cohort. We find that increased years of education is the primary cause of delayed age at first marriage in the youth group, while regionally unique ethnic minority marriage cultures inhibit delayed age at first marriage, and the delaying effect of increased education is stronger than the inhibiting effect of ethnic cultural differences, leading to the result that the age at first marriage is continuously pushed back in the Chinese youth group. In addition, we also find that across the geographic gradient, the more economically developed and open and mobile eastern region has a weakening effect on the postponement of the age at first marriage due to the increase in the average years of education, while the postponement effect is greater in the central region due to the increase in the average years of education of young people, but the increase in the ratio of ethnic minorities strengthens the inhibitory effect of the postponement of the age at first marriage in the eastern region and the central region.

The marginal contribution of this paper lies in the following: firstly, at this stage, the research on the delay of first marriage in China only rests on the results of the sixth census data in 2010, and the research on the phenomenon of first marriage in each province under the data of China's seventh census is still in a blank state. Under the objective reality that Chinese youth are more open, tolerant and autonomous in their views on marriage, the research results in this paper further enrich the timeliness and spatial variability of existing studies on first marriage in China. Second, by focusing on the differences in educational attainment and ethnic culture at the level of geographic gradient, these research results not only allow us to understand the multifaceted and mutually reinforcing relationship of the delayed first marriages of the youth groups, but also provide useful suggestions for alleviating the more prominent conflicts of marital extrusion at the present stage in China.

### Literature review

#### Measurement of age at first marriage.

The measurement of age at first marriage is a topic that has been extensively explored in the literature. This research focuses on two main aspects. The first aspect involves directly measuring the average age at first marriage and comparing the differences. This analysis examines the characteristics of period changes and regional variations in the average age at first marriage across different countries or regions. Additionally, it explores the impact of these differences on subsequent family functions, such as fertility.

During the second demographic transition, both developed and developing countries witnessed a general trend of postponing the mean age at first marriage. It has been observed that an increase in the average age at first marriage negatively affects fertility [15–18]. However, in the context of an aging society, examining only the issue of marriage postponement through the average age at first marriage indicator is not sufficient. This approach fails to consider that individuals with similar average ages at first marriage do not necessarily belong to the same birth cohort. Consequently, the average age at first marriage is influenced by various factors, such as population aging and clustering, which in turn affect the age structure of the population. When comparing the average age at first marriage across different regions, it is important to be aware of bias resulting from the variations in age structures among the populations. As a result, scholars have increasingly focused on exploring the age at first marriage from the perspective of the first marriage pattern.

The second aspect involves constructing marriage tables using census data to examine the evolving characteristics of first marriage patterns and age at first marriage. Marriage tables are generally classified as Gross Nuptiality Tables (GNTs) and Net Nuptiality Tables (NNTs). The gross nuptiality table calculates the expected years in single state by treating the marriage event as a progressively decreasing cohort within the population. However, it fails to consider the influence of the death of unmarried individuals on the age at first marriage. On the other hand, the net nuptiality table considers both the probability of marrying and the likelihood of dying simultaneously by utilizing a life table with a double-decreasing model. By accounting for both marital events and the risk of death among the marriageable population, we obtain data on the probability of marriage, the proportion of unmarried individuals surviving, and the age-specific expected years remaining in the single state. These data remove the influence of population age structure and death risk [19].

In the 1990s and early 2000s, Chinese researchers employed the net nuptiality table method to analyze first marriage patterns in China. They reported that the lifetime marriage rate among Chinese women was nearly 1, indicating a high likelihood of marriage [20]. Additionally, they observed a significant increase in the expected duration of singlehood. By utilizing data on the probability of first marriages, they predicted that the urban‒rural gap in China's future marriage market would gradually widen. The 1985 birth cohort was projected to have a greater proportion of individuals who never married in the future [21]. A study of the population's pattern of first marriages in 2010 revealed a notable decrease in the level of first marriages among females, accompanied by a larger proportion of unmarried males in the population from 1982–2010 [22].

While there has been scarce literature on the pattern of first marriages in the past decade, the period from 2010–2020 is crucial in China's demographic development. The pattern of first marriages among the youth group is undergoing significant transformation. To understand the changes in the age of first marriages by tracking these unions, it is crucial to continue exploring the characteristics of China's first marriage pattern during the second demographic transition. This approach allows us to not only summarize the current stage but also predict future trends in the age at first marriage for young people (woman/man).

### Factors affecting delayed age at first marriage

Factors affecting delayed age at first marriage are influenced by various economic and social factors [22]. Firstly, one key economic factor is the social class identity of individuals and families, which has been extensively researched in relation to marriage matching [23–27]. Over the past fifty years, scholars have increasingly focused on individuals rational decision-making in relation to marriage. They emphasize that individuals weigh the economic status of potential partners and consider whether marriage will result in a better quality of life than remaining single. If the post marriage quality of life is not favorable or offers no benefits, individuals may choose to delay or even forgo marriage [27]. Conversely, if economic resources are sufficient, both partners are more likely to marry early and have more children [28].

In Chinese society, marriage choices often adhere to traditional ideas, with considerations that involve interests exchanged between families of the same social class [29]. Although traditional arranged marriages are explicitly prohibited by Chinese marriage law, young people still seek suitable partners for marriage. “Suitability” is largely determined by factors such as income level, family wealth, and extended family connections [30,31]. An economically distinctive factor of Chinese marriage is the presence of a bride price. Typically, the groom's family pays a significant amount to the bride's family before the wedding, and the bride’s family reciprocates with a symbolic dowry that often serves as start-up capital for the newly formed family. However, in some rural areas, the bride price can be exorbitant, leading many rural mens families to be unable to afford it. Consequently, rural women often opt to marry urban men with higher economic statuses, resulting in an increasing proportion of single rural men [32–34]. In summary, the examination of economic factors in relation to delayed age at first marriage has focused primarily on the rational decision-making of individuals, without fully considering the influence of social policies, culture, and other regional factors on delaying marriage in different areas and time periods.

Secondly, among the social factors, the shift in the concept of marriage and the increase in educational attainment are the primary drivers behind the postponement of marriage. Studies pertaining to the second demographic transition (SDT) attribute the delayed age of marriage largely to changes in individuals and families perceptions of marriage, which serves as the most influential social force distinguishing the second demographic transition from the first [1]. Presently, the global trend entails a delay in the age of first marriage, with East Asian countries such as South Korea and Japan surpassing the rates reported in many Western countries. Notably, even certain African nations are witnessing a significant rise in the age of first marriage [35,36]. While Nordic societies present higher rates of lifelong non-marriage, Chinese residents in East Asian societies still place considerable value on the institution of marriage. Among Chinese urban youth, the transition in the concept of marriage is driven primarily by the pursuit of personal happiness, inspired by increased self-esteem and awareness. Additionally, negative attitudes toward traditional arranged marriages and a weakened authority of elders within families contribute to the ability of youth to make marital choices on the basis of personal preferences [37,38]. Chinese scholars have focused predominantly on the endogenous dynamics underlying the changing concept of marriage among contemporary youth. They analyze its impact on the postponement of marriage from the perspective of family authority and intergenerational transmission.

Moreover, the delay in educational attainment has led to shifts in gender roles within society. In traditional societies, men are the primary breadwinners, whereas women typically assume caregiving and child-rearing responsibilities [39]. As women gain greater economic independence in modern society, early marriage for financial stability is no longer necessary. This change is largely attributed to improvements in women's education levels and social status [40,41]. The “Action Plan for Revitalization of Education for the 21st Century [42]” issued by China in 1999 expanded access to nine-year basic education to over 80% of the population and significantly increased enrollment in higher education. This policy has influenced the age of first marriage, particularly among highly educated Chinese youth, who are more prone to remain unmarried. The impact is more pronounced for women, and data suggest that women over the age of 30 with higher education face greater challenges in establishing successful marriages [43–46]. In summary, the examination of social factors contributing to the postponement of marriage primarily focuses on the influence of specific indicators. However, it remains unclear whether other factors play a dominant role in shaping the delay in first marriage among Chinese youth in the 21st century.

### Spatial differences in delayed age at first marriage

The delay in the age at first marriage among Chinese youth displays significant regional variations, driven by economic differentiation and social stratification. At the macro level, differences between urban and rural areas, as well as gender disparities, are evident. These differences are characterized mainly by the continual convergence of the probability of first marriage between males and females, alongside a prevalent trend of late marriage and non-marriage among rural males [47]. For example, over the past 30 years, the likelihood of first marriage for young women has decreased significantly, aligning with the age range typical for men. However, the proportion of lifelong unmarried men surpasses that of women. The gender imbalance in the birth cohort from 1984--2004, resulting in an 18 million shortfall of female babies compared with males in China—and the phenomenon of rural‒urban intermarriages further contributed to the marriage squeeze among rural males. Consequently, rural men have a lower probability and later expected duration of singlehood than their urban counterparts do [48]. This situation presents the most prominent issue in the urban‒rural disparity of first marriage patterns in China today. At the micro level, class differences accentuate the problem, specifically the conflict between the upper class's internal circulation of marriage resources and the aspirations of the middle and lower classes for upward mobility. In developed areas, some rural women find it challenging to attain upward mobility through marriage resources while being unwilling to accept similar or lower-class resources, resulting in consistent delays in their age at first marriage. On the other hand, disadvantaged males from the lower class seek partnerships with women from less developed or underdeveloped regions to alleviate their marital pressures. This imbalance in the transmission of marital pressure ultimately leads to the predicament faced by lower stratum males from less developed or underdeveloped areas, where they struggle to find marriage prospects or remain unmarried indefinitely. However, existing research on the age at first marriage among Chinese youth has focused predominantly on overall trends and urban‒rural and gender differences, failing to examine the differentiating characteristics and influential factors of delayed age at first marriage at the provincial level.

To summarize, existing research has concluded that the delay in the age of first marriage for young Chinese people is an inevitable process during the second demographic transition and that it will persist over the long term. There are many factors affecting the delay in age at first marriage, of which economic and social factors are the main ones. However, at present, there are obvious inter-provincial differences in the scene of first-marriage delays among youth groups brought about by China's unique dual urban-rural structure, the unevenness of regional demographic development, and the shift in the culture of marriage and childbearing. Under the reality of Chinese youth's more open, tolerant and autonomous view of marriage, it is necessary to explore whether the spatial and temporal differences in the completion of first marriage among youth groups are gradually widening from the provincial administrative scales with distinctive heterogeneity of marriage culture. Is there a similar trend in the change of age at first marriage between the whole and the parts of the country? To explore whether the factors and mechanisms influencing the delay in age at first marriage are more diversified and mutually reinforcing? These are the scientific questions that need to be addressed.

Therefore, this paper utilizes the data from China's three population censuses from 2000 to 2020 and the Western Region Life Tables published by the United Nations Population Division to analyze the gender, urban-rural, and regional differences in the pattern of first marriages in each province of China in 2020 by using the Multi-decreasing Table to compare and analyze the characteristics of the changing pattern of first marriages in China since the 21st century. We also utilize the 1996–2020 China Statistical Yearbook data to construct the panel data of 31 provinces in China for 25 years, to explore the influencing factors of the delayed age at first marriage through the dual fixed-effects model of region and time, and to explore the core driving mechanism of the delayed age at first marriage in China at this stage, with a view to making reasonable explanations and useful references for the special phenomenon of delayed age at first marriage in the second demographic transition of China which is different from that of the developed countries. This is to provide a reasonable explanation and useful reference for the special phenomenon of delayed first marriage in China's second demographic transition, which is different from that of developed countries.

## Methods and data

### Data sources

To construct the Net Nuptiality Table for different provinces, it is necessary to refer to the data on first marriages and the number of deaths among the unmarried population in each province. This study drew on established methodological approaches by leveraging mortality probabilities from the United Nations West Model Life Table for developing countries and used province-specific life expectancy by sex as the reference to derive proxy mortality rates for never-married populations [49–53]. We also leveraged urban-rural/age/sex/marriage-year-specific population data from the 2000–2020 population censuses to compute first-marriage probabilities, expected years in single state, and proportions remaining unmarried. It should be clarified that the number of age-specific first marriage was derived from marriage-year/age/sex tabulations in Volume 5 of China's census long-form micro-data (2000, 2010 and 2020). Since the 2020 census enumeration concluded in November 2020 and thus did not achieve full calendar-year coverage, we utilized 2019 data to ensure complete annual representation in nuptiality table construction.

To study the impact mechanism of delayed age at first marriage, this paper focuses on the period from 1996--2020, taking into account data availability and the concentrated period of China's second demographic transition. A total of 775 balanced panel data points is collected for the 31 Chinese provinces (excluding Hong Kong, Macao, and Taiwan). The base map used in Fig 3 was sourced from the Standard Map Service System of the National Geomatics Center of China [54].

**Fig 1:**
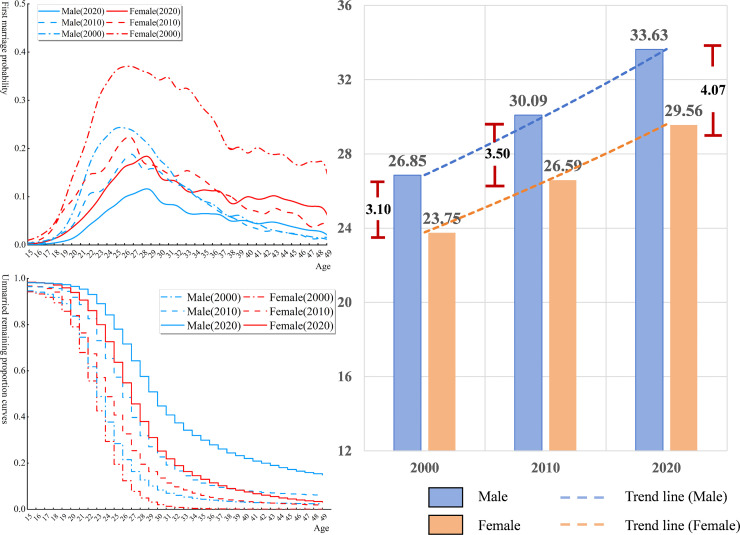
The first marriage pattern of young people by gender in China from 2000 to 2020: (a) First Marriage probability, (b) Unmarried Remaining proportion curves and (c) the expected duration of singlehood since birth.

**Fig 2:**
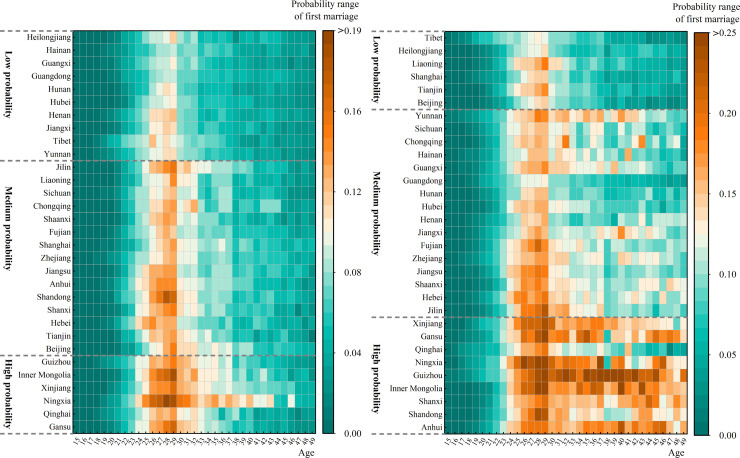
First marriage probability in different provinces of China: young men and young women in 2020.

### Variables selection

The dependent variable used was the average age at first marriage. This indicator reflected the average age at which first marriages occurred within a given year in a specific country or area. In this paper, the average age at first marriage by gender in 31 provinces from 1996 to 2020 was selected, with a sample size of 775. The average age at first marriage for the overall Chinese sample increased from 24.15 to 28.67 years old, a delay of nearly five years. It characterized the age structure of the population and the trend of age at first marriage during population development.

Independent variables included education and ethnic differences. The education variable was defined as the years of education, measured by the average number of years of education, which was calculated using the number of individuals in each year of education and the duration of each level, as indicated in the statistical yearbook. Ethnic differences were represented by the ethnic minority ratio, which indicated the proportion of ethnic minorities to the total population and reflected the cultural attributes of the region.

To address the issues of covariance and endogeneity in the measurement model, as well as to account for differences in economic development and population age structure across provinces and regions, three control variables were selected on the basis of the relevant literature [55–58]. The control variables chosen included regional development, living standards, and age structure. Specifically, regional development was measured by the proportion of the urban population to the total population, residents’ living standards were measured by per capita disposable income, and the population age structure was measured by the all-age dependency ratio, which reflected the burden of the population.

### Net nuptiality table

Similar to life tables, marriage tables are a method used to calculate the expected years in single state by treating marital events as progressively decreasing forms within a population cohort. Net Nuptiality tables, on the other hand, are multi-decreasing life tables that consider both the probability of marrying and the probability of dying. This approach eliminates the influence of population age structure and mortality risk when calculating the probability of marriage, the proportion of unmarried individuals still alive, and the age-specific expected years remaining in the single state. In constructing the multi-decreasing first marriage table, reference was made to existing research findings [57], which identified that first marriages predominantly occur between exact ages 15 and 49. The process of constructing the multi-decreasing first marriage table is provided below:

First, the age-specific probability of first marriage $\;n_{x}\;$ and the age-specific probability of death $q_{x}\;$ in the multi-decreasing first marriage table need to be computed separately via the following formulas [53,59]:


$$n_{x}=\frac{2\mu_{x}}{2+\mu_{x}}$$ (1)

$$q_{x}=\frac{2m_{x}}{2+m_{x}}$$ (2)

where $n_{x}\;$ is the probability of first marriage at exact age *x*, converted from the rate of first marriages during the year $\mu_{x}$ during the year, and where $q_{x}\;$ is the probability of death at exact age *x*, estimated from the age-specific unmarried mortality rate $m_{x}\;$. Because of the lack of statistics on the marital status of those who die, we usually use the probability of death in the year as a proxy when we assume that the occurrence of death is unrelated to the occurrence of marriage. To save space in the manuscript, the derivation process of the Details about the probability of first marriage is presented in Supporting Information S1.

Second, the number of unmarried survivors $l_{x}$, the number of unmarried deaths $d_{x}\;$, and the number of first marriages $f_{x}$ are further computed [60–62]. In a cohort of people born at the same time, since the number of first marriages and the number of unmarried deaths decreases with age, the academic community usually defines the initial number of people in the multi-decreasing first marriage table as *100,000* at the age of *0*, i.e., $l_{\text{0}}=\text{1}\text{0}\text{0}\text{0}\text{0}\text{0}$, and the formula is as follows:


$$l_{x}=\left\{ \begin{array}{c} l_{x-1}-d_{x-1}=l_{x-1}-l_{x-1}q_{x-1}\text{for\textbackslash{} }x\leq 15\\ l_{x-1}-f_{x-1}-d_{x-1}=l_{x-1}-l_{x-1}n_{x-1}-l_{x-1}q_{x-1}\text{for\textbackslash{} }15<x<50 \end{array}\right.$$ (3)

In Equation (3), the probability of a first marriage occurring before the exact age of 15 is extremely low; thus, at this stage, the number of unmarried survivors $l_{x}$ is also the number of remaining survivors, and only the diminishing effect of the number of deaths needs to be taken into account. It should be noted that the model specified in Equation (3) does not explicitly include two likelihoods: (a) to marry and then die within the same age interval, and (b) to die while about to get married. These were omitted following the common practice in nuptiality table construction, as the simultaneous estimation of these joint events requires detailed data that are typically unavailable. Given the very low mortality rates prevalent among the marriageable-age population in China, the exclusion of these joint events does not introduce substantial bias and is not expected to significantly affect the results for academic research purposes. This simplification is considered acceptable in demographic studies when focusing on marriage patterns rather than precise actuarial calculations [63]. Starting from exact age 15 up to exact age 49, the number of unmarried survivors $l_{x}$ needs to consider both the effects of death and first marriage, where $f_{x}$ is obtained from the product of the probability of the first marriage $n_{x}$ in Equation (1) and the number of unmarried survivors $l_{x}$.

The average number of unmarried person-years lived by the marriageable cohort between exact ages 15 and 49 who remain unmarried until age x is calculated as $L_{x}$:


$$L_{x}=l_{x}+\frac{l_{x}-l_{x-1}}{\text{2}}$$ (4)

Notably, we assume that there are no more first marriages in the open age group from exact age 50 and above; thus, the average number of unmarried person-years in the corresponding first-marriage table is the number of deaths, $L_{\text{5}\text{0}+}$, and by bringing the average number of unmarried person-years in each age group into Equation (5), the total number of unmarried person-years, $T_{x}$, at age *x* can be further calculated:


$$T_{x}=\sum_{i=x}^{50+}L_{x}$$ (5)

Finally, we can find the average number of years that the unmarried surviving population at exact age *x* has been in the event of a first marriage, i.e., the expected years in the single state at age *x*, $e_{x}$:


$$e_{x}=\frac{T_{x}}{l_{x}}$$ (6)

To show more clearly and visually the age at which the first marriage event occurs, we convert the expected years in single state $e_{\text{1}\text{5}}$ at exact age 15 to the expected duration of singlehood since birth ${fma}_{\text{0}}$:


$$fma_{\text{0}}=e_{15}+15$$ (7)

The complete Net nuptial table in Supporting Information S2.

### Fixed effect model

Using balanced panel data for 31 provinces from 1996--2020, we conducted an analysis to identify the factors influencing the delay in the age of first marriage in China as a whole and by gender. The formulas used are shown below:


$$Y_{it}=E_{it}\beta_{\text{1}}+R_{it}\beta_{\text{2}}+X_{it}\beta_{\text{3}}+\gamma_{i}+\alpha_{i}+u_{it}$$

where $Y_{it}$ represents the logarithm of the average age at first marriage in year *t* in province *i*, $E_{it}$ represents the logarithm of the average years of schooling in the corresponding year and province, $R_{it}$ represents the level of ethnocultural differences in the corresponding year and province, $X_{it}$ corresponds to the relevant control variable, $\gamma_{i}$ represents the area fixed effect in province *i*, $\alpha_{i}$ represents a dummy variable for year *t*, and $u_{it}$ denotes the randomized disturbance term. Compared with OLS regression estimation, the use of a fixed-effects model with time dummy variables allows for simultaneous control of the effects of time-varying regional factors that do not vary over time and macro factors that do not vary over time on the average age at first marriage.

## Results

### Expected pre-marital singlehood duration

The overall probability of first marriage for females was generally higher than that for males (Fig 1a). The highest first marriage probability for both genders occurred in 2000, and the lowest in 2020. The peak first marriage probabilities for males and females were gradually converging, and the age at which the highest first marriage probability occurred was continuously being delayed. The proportion of unmarried individuals showed that in 2000, the proportion of unmarried females at age 49 was nearly 0%, while in 2020, it had increased to 3.9% (Fig 1b). In contrast, the proportion of unmarried males at age 49 was 2.3% in 2000, and by 2020, it had risen to 15.3%. The expected years in single state for both males and females in China had steadily increased (Fig 1c). In 2000, the expected of duration singlehood since birth for males was 26.85 years, and for females, it was 23.75 years, with a gap of 3.1 years. By 2020, the expected duration for males had risen to 33.63 years, and for females, it had reached 29.56 years, with the gap widening to 4.07 years.

Using cluster analysis, we categorized first-marriage probabilities by gender into low, medium, and high regional groupings (Fig 2). Females consistently showed higher first-marriage probabilities than males across age groups. The peak probability for males occurred at ages 26–30, with lower-probability provinces concentrated in central and southwestern China, while higher-probability clusters predominated in ethnic minority regions. For females, high-probability ages began at 24, declining after 30. Female low-probability provinces (predominantly in northeast China and eastern municipalities) were fewer than male counterparts, whereas high-probability areas extended beyond ethnic regions to include coastal (e.g., Shandong) and central provinces (e.g., Shanxi, Anhui).

**Fig 3:**
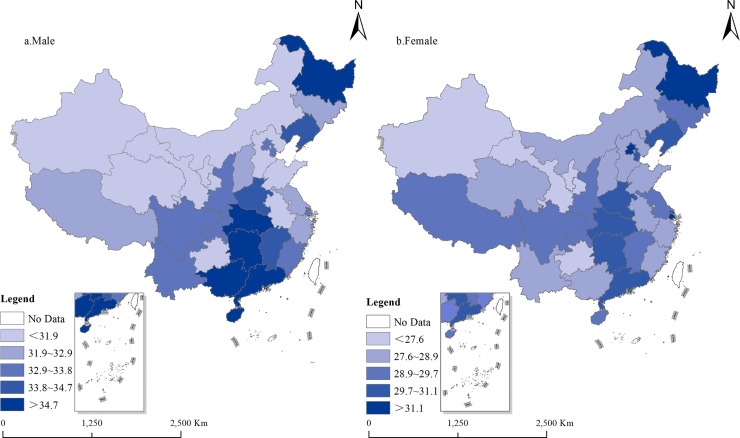
Number of unmarried years experienced by young people in 2020. Note: The base map used in Fig 3 was sourced from the Standard Map Service System of the National Geomatics Center of China (accessed January 19, 2025, available at: http://bzdt.ch.mnr.gov.cn/). Cartographic software: ArcGis 10.8.

In 2020, the expected duration of singlehood since birth was generally higher for males than for females (Fig 3). In the southern region, males were the latest (greater than 34.7 years). In the northern region, except for Heilongjiang, Jilin, and Liaoning, males were generally lower than 31.9 years. However, females in the municipalities were the highest (greater than 31.1 years). Provinces with a lower expected years in the single state for females were concentrated in the western of Xinjiang, Gansu, Qinghai, Ningxia, and the central of Inner Mongolia.

**Fig 4:**
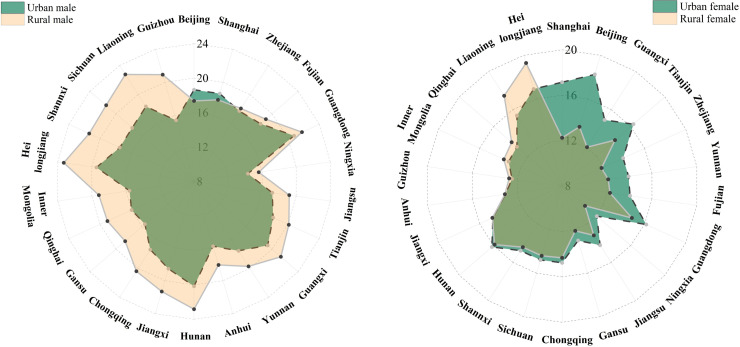
Urban–rural comparison of the number of years of unemployment experienced at aged 15.

Our analysis of urban-rural disparities in expected years in the single state since birth by gender revealed distinct patterns: urban males exhibited fewer expected unmarried years than rural males nationwide, except in Beijing and Shanghai where this trend reversed (Fig 4). Conversely, urban females demonstrated greater unmarried expectancy than rural females in over half of provinces, with the most pronounced gaps in affluent eastern metropolises (Beijing, Shanghai, Tianjin). These spatial patterns reflect prolonged singlehood years among China's 2020 cohort with widening gender gaps, particularly concentrated in narrowing age windows for males. The persistence of fewer singlehood years among urban males and rural females contrasts sharply with delayed transitions among urban females in developed regions and rural males in disadvantaged areas, necessitating investigation of underlying mechanisms and key factors shaping these differentials.

### Descriptive statistics and regression results

Table 1 showed that the descriptive statistics results for the selected variables. The standard deviation of the average age at first marriage, both for the overall sample and by gender, is small. The mean age at first marriage for the total sample is 25.33, 26.32 for males, and 24.44 for females. The average years of education for the total sample is 8.26 years, 9.57 years for males, and 7.82 years for females.

**Table 1:** Variable definitions and descriptive statistics in 1997–2020 (n = 775).

Variable	definition	Male	Female	Total
		Mean (Std)	Mean (Std)	Mean (Std)
First Marriage Age (*Y*_*it*_)		26.32 (0.05)	24.44(0.06)	25.33(0.06)
Education	Average years of schooling	9.57 (0.16)	7.82(0.22)	8.26 (0.19)
Ethnic Differences	Ethnic minorities in total population	0.16(0.22)	0.16 (0.22)	0.16(0.22)
Living Standards	Log of Per capita disposable income	9.44(0.65)	9.44(0.65)	9.44(0.65)
Regional Development	Urbanization rate	0.48(0.17)	0.48(0.17)	0.48(0.17)
Age Structure	Age dependency ratio	39.77 (7.92)	39.76(7.92)	39.76(7.92)

After controlling for regional variations, each additional year of educational attainment delayed the age at first marriage by 0.22 years (β = 0.219, p < 0.01; Table 2), indicating that higher education prolongs singlehood through extended schooling and career establishment. Conversely, although the effect of ethnic minority proportion was statistically significant at both national (β = −0.041, p < 0.05) and female-specific levels (β = −0.037, p < 0.05), its demographic impact was negligible.

**Table 2:** Area fixed effects model regression results: total sample, male sample and female sample in 1997-2020 (n = 775).

Var	(1)	(2)	(3)
	*Fixed*	*Fixed*	*Fixed*
	Total	Male	Female
Education	0.219^***^	0.158^***^	0.159^***^
	(0.038)	(0.045)	(0.015)
Ethnic Differences	−0.041^**^	−0.009	−0.037^**^
	(0.019)	(0.025)	(0.168)
Living Standards	0.000^***^	0.000^***^	0.000^***^
	(0.000)	(0.000)	(0.000)
Regional Development	0.121^**^	0.139^**^	0.142^***^
	(0.043)	(0.053)	(0.017)
Age Structure	0.003^***^	0.003^***^	0.004^***^
	(0.000)	(0.000)	(0.000)
Cons	2.547^***^	2.713^***^	2.617^***^
	(0.077)	(0.092)	(0.084)
Fixed effects for regions	Yes	Yes	Yes
N	775	775	775
R-squared	0.579	0.474	0.528

Robust standard error is reported in parentheses in the table, and this model is the area fixed effects with clustering robust standard error result; ^***^, ^**^, and ^*^ denote statistical significance at the < 0.01, < 0.05, and <0.1 levels, respectively

After incorporating temporal effects via regional fixed effects (Table 3), although educational attainment and ethnic minority composition remained significant predictors of first-marriage timing, the effect size of education on age at first marriage was significantly reduced (β = 0.066, p < 0.01 for males; β = 0.062, p < 0.01 for females). Similarly, despite statistical significance, the impact of ethnic minority proportion was demographically negligible at the national level (β = −0.022, p < 0.05 for females).

**Table 3:** Two-factor fixed effects model regression results: total sample, male sample and female sample in 1997-2020 (n = 775).

Var	(4)	(5)	(6)
	*Fixed*	*Fixed*	*Fixed*
	Total	Male	Female
Education	0.103^***^	0.066^***^	0.062^**^
	(0.019)	(0.019)	(0.022)
Ethnic Differences	−0.026^**^	−0.027	−0.022**
	(0.01)	(0.018)	(0.011)
Living Standards	0.000^*^	0.000	0.000^***^
	(0.000)	(0.000)	(0.000)
Regional Development	0.023	0.012	0.038
	(0.027)	(0.037)	(0.029)
Age Structure	0.001^***^	0.002^***^	0.001^***^
	(0.000)	(0.000)	(0.000)
Cons	2.93^***^	3.053^***^	2.971^***^
	(0.049)	(0.048)	(0.051)
Fixed effects for regions	Yes	Yes	Yes
Fixed effects for time	Yes	Yes	Yes
N	775	775	775
R-squared	0.902	0.889	0.91
Prob>F = 0.000			

The Global Moran's Index was no significant spatial autocorrelation of the age at first marriage at the provincial level in China (see Supporting Information S3). Considering the differences in education levels and ethnic cultures in China, we divided three regions (i.e., Eastern, Central, and Western, See Supporting Information S4) for the analysis of how the average years of education and ethnic culture influenced the age at first marriage. The Eastern included Beijing, Tianjin, Hebei, Liaoning, Shanghai, Jiangsu, Zhejiang, Fujian, Shandong, Guangdong, and Hainan. The Central included Shanxi, Inner Mongolia, Jilin, Heilongjiang, Anhui, Jiangxi, Henan, Hubei, Hunan, and Guangxi. The Western included Chongqing, Sichuan, Guizhou, Yunnan, Tibet, Shaanxi, Gansu, Qinghai, Ningxia, and Xinjiang. The results of the influence of education on the age at first marriage (Table 4) showed that the delaying effect of average years of education on the age at first marriage was weaker in the Eastern compared to other regions (−0.052, P ≤ 0.01), while in the Central, this effect was stronger (0.07, P ≤ 0.01). No significant correlation was found in the Western. Additionally, the regional differences in the age at first marriage were consistent for young males and young females.

**Table 4:** The analysis of regional differences between education and age at first marriage.

Variables	Coefficients (St. Err)								
	Total (1)			Male (2)			Female (3)		
	East	Central	West	East	Central	West	East	Central	West
Edu	0.103^***^	0.109^***^	0.129^***^	0.064^***^	0.067^***^	0.093^***^	0.063^***^	0.067^***^	0.074^***^
	(0.016)	(0.016)	(0.024)	(0.014)	(0.014)	(0.025)	(0.013)	(0.013)	(0.019)
Edu × region	−0.052^***^	0.07^***^	−0.023	−0.06^***^	0.074^***^	−0.027	−0.045^***^	0.054^***^	−0.011
	(0.016)	(0.015)	(0.016)	(0.02)	(0.018)	(0.019)	(0.013)	(0.013)	(0.013)
Control Variables					Yes				
Fixed effects for regions & years					Yes				

The results of the influence of ethnic culture on the age at first marriage (Table 5) showed that in the Eastern and Central, an increase in the proportion of ethnic minorities would weaken the delaying effect on the age at first marriage (East: −0.957, P ≤ 0.01; Central: −0.586, P ≤ 0.01). In the Western, however, an increase in the proportion of ethnic minorities would strengthen the delaying effect on the age at first marriage (0.07, P ≤ 0.01). Additionally, both males and females exhibited characteristics consistent with the national overall trend.

**Table 5:** The analysis of regional differences between ethnic and age at first marriage.

Variables	Coefficients (St. Err)								
	Total (1)			Male (2)			Female (3)		
	East	Central	West	East	Central	West	East	Central	West
Ethnic	−0.022^**^	−0.023^**^	−0.769^***^	−0.023^***^	−0.023^***^	−0.736^***^	−0.018	−0.019	−0.716^***^
	(0.012)	(0.012)	(0.118)	(0.012)	(0.012)	(0.119)	(0.012)	(0.012)	(0.124)
Eth × region	−0.957^***^	−0.586^***^	0.749^***^	−0.944^***^	−0.541^***^	0.716^***^	−1.004^***^	−0.477^***^	0.7^***^
	(0.188)	(0.153)	(0.118)	(0.19)	(0.154)	(0.119)	(0.198)	(0.161)	(0.125)
Control Variables					Yes				
Fixed effects for regions & years					Yes				

On the other hand, when the minority ratio is considered, an increase in the proportion of minorities in eastern and central China has a strengthening effect on the reduction in the age at first marriage, whereas the western region has a weakening trend. This can be explained by similar characteristics observed in the average years of schooling. Specifically, the western region is home to a large number of China's ethnic minorities, which constitute a greater proportion of the total population than other regions do. Additionally, the western region includes the most diverse range of ethnic minorities. Consequently, when focusing solely on the impact of the ethnic minority ratio on the delayed age at first marriage in the western region, the similarity in their ethnic minority marriage cultures diminishes the effect it produces.

Further analysis of the regional heterogeneity of the influence mechanism on age at first marriage reveals consistent characteristics in the Chinese male and female cohorts across the geographic gradient. The average years of schooling have a weakening effect on delaying first marriage in the eastern region but a strengthening effect in the central region for both males and females. This can be attributed to the higher level of economic development and social and cultural openness in the eastern region, which provides more opportunities for young people to receive higher education and vocational training. In addition to individual education level, other social factors, such as competitive preferences and high real estate prices, also contribute to weakening the effect of education on age at first marriage postponement, to some extent. Conversely, the central region has weaker educational resources, more conservative economic development, and cultural openness, which makes educational attainment more crucial for the social status and development expectations of the different group, resulting in a stronger effect of education on age at first marriage postponement than in the eastern region. Moreover, the ethnic minority ratio weakens the delay in the age at first marriage in the western region but strengthens it in both the eastern and central regions.

## Discussion

Our provincial-level Net Nuptiality Table analysis revealed significant shifts in unmarried expectancy across China between 2000 and 2020. The expected years in the single state since birth increased from 26.85 to 33.63 years for males and from 23.75 to 29.56 years for females, while the gender gap in singlehood duration widened from 3.1 to 4.7 years. Geographically, southern provinces recorded higher unmarried expectancy for males compared to western regions with large ethnic minority populations, whereas eastern municipalities had longer singlehood years for females than provinces like Xinjiang and Gansu. Urban-rural disparities varied: in municipalities, urban males experienced slightly longer unmarried periods than rural males, but the opposite occurred in non-municipal provinces. Urban females consistently showed higher unmarried expectancy than rural females nationwide, with the most pronounced gaps observed in economically developed areas.

Further analysis of the influence of educational and ethnic culture on the age at first marriage revealed that the average years of education were positively correlated with the age at first marriage after controlling for regional and temporal effects, while the proportion of ethnic minorities were negatively. Considering regional differences, no significant spatial autocorrelation of the provincial-level age at first marriage was found. However, there were interactive differences in the relationship between educational attainment, ethnic culture, and age at first marriage in the eastern, central, and western. We found that in the eastern, the impact of average years of education on the age at first marriage was diminishing, while in the central, it had an enhancing effect. The proportion of ethnic minority status had a diminishing effect on the delay of the age at first marriage in the eastern and central, but an enhancing effect in the western.

Previous studies found that in Asian countries, the age at first marriage was delayed due to higher educational levels. A study in Japan showed that higher university education was strongly associated with later and fewer marriages [59]. A qualitative study in India revealed that marriage often interrupted the education phase [60]. Our research also found similar results. The delay in the age at first marriage was significantly related to the increase in average years of education. From a personal life cycle perspective, this phenomenon suggested that young people in the education stage lacked financial independence compared to those in the employment stage, as they still relied on their families of origin for living costs [61]. On the other hand, it could be interpreted as a cultural trait in East Asian countries, where marriage was seen as a step to be taken after achieving social status [64].

A study in Ghana found that minority ethnic groups were associated with early marriage, with regional differences [65]. Our study also revealed similar findings, although this effect exhibited a weaker influence on first marriage age in China. The increase in the proportion of minority ethnic groups was negatively correlated with the age at first marriage. In the eastern and central regions of China, a higher proportion of minorities also decreased the trend of later age at first marriage. A possible explanation was that marriage attitudes in minority groups were more traditional compared to the Han. Some young people in intermarriage might be influenced by these traditional attitudes and entered marriage at an earlier age [66].

Although our study calculated the expected years in single state at the provincial level and explored the relationship between education, ethnic minority, and age at first marriage. However, there were also some limitations. First, due to data limitations on age-specific mortality rates, our study could not calculate the expected duration of singlehood for consecutive years. As a result, the dependent variable in our regression analysis was replaced by the average age at first marriage. We were uncertain whether factors like birth rates and mortality rates, which affect population reproduction, were related to the age at first marriage. Second, due to data availability, our study was unable to obtain cohort data on age-specific first marriage populations and unmarried population mortality data by province in China. Instead, we used point-in-time data from the census as a proxy for the hypothetical cohort and mortality data from the West model life tables for the calculations. Third, Provincial-level aggregation likely masks critical sub-provincial heterogeneities, including urban-rural disparities in marriage markets, localized cultural/economic variations (e.g., ethnic enclaves), and uneven educational infrastructure. This spatial simplification risks ecological fallacy when interpreting provincial results, as averaged data may not reflect internal regional dynamics.

## Conclusion

Our study examines spatial-temporal variations in expected years in single state across demographic groups in China. Over the past two decades, the expected duration of singlehood has demonstrated consistent nationwide growth. By 2020, males averaged 33.68 expected years in single state, while females registered 29.16 years. Gender disparities in singlehood duration revealed divergent urban-rural trajectories. Educational attainment emerged as the primary factor associated with marital timing delays. Higher average education years showed significant positive associations with first marriage age, whereas ethnic minority proportions exhibited statistically weak negative correlations. Regional heterogeneity was observed in these associations: Central China manifested stronger educational effects on marital delays than other regions, while increased ethnic minority concentration mitigated first marriage postponement in both eastern and central China.
